# Cerebrovascular disease is associated with Alzheimer’s plasma biomarker concentrations in adults with Down syndrome

**DOI:** 10.1093/braincomms/fcae331

**Published:** 2024-09-25

**Authors:** Natalie C Edwards, Patrick J Lao, Mohamad J Alshikho, Olivia M Ericsson, Batool Rizvi, Melissa E Petersen, Sid O’Bryant, Lisi Flores Aguilar, Sabrina Simoes, Mark Mapstone, Dana L Tudorascu, Shorena Janelidze, Oskar Hansson, Benjamin L Handen, Bradley T Christian, Joseph H Lee, Florence Lai, H Diana Rosas, Shahid Zaman, Ira T Lott, Michael A Yassa, Alzheimer’s Biomarkers Consortium–Down Syndrome (ABC-DS) Investigators, Howard J Aizenstein, Beau M Ances, Howard F Andrews, Karen Bell, Rasmus M Birn, Adam M Brickman, Peter Bulova, Amrita Cheema, Kewei Chen, Bradley T Christian, Isabel Clare, Ann D Cohen, John N Constantino, Eric W Doran, Natalie C Edwards, Anne Fagan, Eleanor Feingold, Tatiana M Foroud, Benjamin L Handen, Jordan Harp, Sigan L Hartley, Elizabeth Head, Rachel Henson, Christy Hom, Lawrence Honig, Milos D Ikonomovic, Sterling C Johnson, Courtney Jordan, M Ilyas Kamboh, David Keator, William E Klunk, Julia K Kofler, William Charles Kreisl, Sharon J Krinsky-McHale, Florence Lai, Patrick Lao, Charles Laymon, Joseph H Lee, Ira T Lott, Victoria Lupson, Mark Mapstone, Chester A Mathis, Davneet Singh Minhas, Neelesh Nadkarni, Sid O’Bryant, Melissa Parisi, Deborah Pang, Melissa Petersen, Julie C Price, Margaret Pulsifer, Michael S Rafii, Eric Reiman, Batool Rizvi, Herminia Diana Rosas, Laurie Ryan, Frederick Schmitt, Nicole Schupf, Wayne P Silverman, Dana L Tudorascu, Rameshwari Tumuluru, Benjamin Tycko, Badri Varadarajan, Desiree A White, Michael A Yassa, Shahid Zaman, Fan Zhang, José Gutierrez, Donna M Wilcock, Elizabeth Head, Adam M Brickman

**Affiliations:** Taub Institute for Research on Alzheimer’s Disease and the Aging Brain, Columbia University, New York City, NY 10032, USA; Department of Neurology, Vagelos College of Physicians and Surgeons, Columbia University, New York City, NY 10032, USA; Department of Neuroscience, Columbia University, New York City, NY 10032, USA; Taub Institute for Research on Alzheimer’s Disease and the Aging Brain, Columbia University, New York City, NY 10032, USA; Department of Neurology, Vagelos College of Physicians and Surgeons, Columbia University, New York City, NY 10032, USA; Taub Institute for Research on Alzheimer’s Disease and the Aging Brain, Columbia University, New York City, NY 10032, USA; Department of Neurology, Vagelos College of Physicians and Surgeons, Columbia University, New York City, NY 10032, USA; Taub Institute for Research on Alzheimer’s Disease and the Aging Brain, Columbia University, New York City, NY 10032, USA; Department of Neurology, Vagelos College of Physicians and Surgeons, Columbia University, New York City, NY 10032, USA; Department of Neurobiology & Behavior, University of California, Irvine, CA 92697, USA; University of North Texas Health Science Center, Department of Pharmacology and Neuroscience, Fort Worth, TX 76107, USA; University of North Texas Health Science Center, Department of Pharmacology and Neuroscience, Fort Worth, TX 76107, USA; Department of Pathology and Laboratory Medicine, University of California Irvine School of Medicine, University of California, Irvine, CA 92617, USA; Taub Institute for Research on Alzheimer’s Disease and the Aging Brain, Columbia University, New York City, NY 10032, USA; Department of Neurology, Vagelos College of Physicians and Surgeons, Columbia University, New York City, NY 10032, USA; Department of Neurology, University of California, Irvine, CA 92697, USA; Department of Psychiatry, University of Pittsburgh, Pittsburgh, PA 15213, USA; Clinical Memory Research Unit, Department of Clinical Sciences Malmö, Lund University, Lund 221 00, Sweden; Clinical Memory Research Unit, Department of Clinical Sciences Malmö, Lund University, Lund 221 00, Sweden; Memory Clinic, Skåne University Hospital, Malmö 214 28, Sweden; Department of Psychiatry, University of Pittsburgh, Pittsburgh, PA 15213, USA; Waisman Center, University of Wisconsin-Madison, Madison, WI 53705, USA; Taub Institute for Research on Alzheimer’s Disease and the Aging Brain, Columbia University, New York City, NY 10032, USA; Department of Neurology, Vagelos College of Physicians and Surgeons, Columbia University, New York City, NY 10032, USA; Department of Neurology, Harvard Medical School, Massachusetts General Hospital, Boston, MA 02114, USA; Department of Neurology, Harvard Medical School, Massachusetts General Hospital, Boston, MA 02114, USA; Department of Radiology, Center for Neuroimaging of Aging and Neurodegenerative Diseases, Athinoula A. Martinos Center for Biomedical Imaging, Charlestown, MA 02129, USA; Department of Psychiatry, University of Cambridge, Cambridge CB2 0SZ, UK; Department of Pediatrics and Neurology, School of Medicine, University of California, Irvine, CA 92868, USA; Department of Neurobiology & Behavior, University of California, Irvine, CA 92697, USA; Center for the Neurobiology of Learning and Memory, University of California, Irvine, CA 92697, USA; Department of Neurology, Vagelos College of Physicians and Surgeons, Columbia University, New York City, NY 10032, USA; Stark Neurosciences Research Institute, Indiana University School of Medicine, Indianapolis, IN 46202, USA; Department of Neurology, Indiana University School of Medicine, Indianapolis, IN 46202, USA; Department of Anatomy, Cell Biology and Physiology, Indiana University School of Medicine, Indianapolis, IN 46202, USA; Department of Pathology and Laboratory Medicine, University of California Irvine School of Medicine, University of California, Irvine, CA 92617, USA; Taub Institute for Research on Alzheimer’s Disease and the Aging Brain, Columbia University, New York City, NY 10032, USA; Department of Neurology, Vagelos College of Physicians and Surgeons, Columbia University, New York City, NY 10032, USA

**Keywords:** Alzheimer’s disease, Down syndrome, cerebrovascular disease, magnetic resonance imaging, biomarkers

## Abstract

By age 40 years, over 90% of adults with Down syndrome have Alzheimer’s disease pathology and most progress to dementia. Despite having few systemic vascular risk factors, individuals with Down syndrome have elevated cerebrovascular disease markers that track with the clinical progression of Alzheimer’s disease, suggesting a role of cerebrovascular disease that is hypothesized to be mediated by inflammatory factors. This study examined the pathways through which small vessel cerebrovascular disease contributes to Alzheimer’s disease-related pathophysiology and neurodegeneration in adults with Down syndrome. One hundred eighty-five participants from the Alzheimer’s Biomarkers Consortium–Down Syndrome [mean (SD) age = 45.2 (9.3) years] with available MRI and plasma biomarker data were included in this study. White matter hyperintensity (WMH) volumes were derived from T2-weighted fluid-attenuated inversion recovery MRI scans, and plasma biomarker concentrations of amyloid beta 42/40, phosphorylated tau 217, astrocytosis (glial fibrillary acidic protein) and neurodegeneration (neurofilament light chain) were measured with ultrasensitive immunoassays. We examined the bivariate relationships of WMH, amyloid beta 42/40, phosphorylated tau 217 and glial fibrillary acidic protein with age-residualized neurofilament light chain across Alzheimer’s disease diagnostic groups. A series of mediation and path analyses examined statistical pathways linking WMH and Alzheimer’s disease pathophysiology to promote neurodegeneration in the total sample and groups stratified by clinical diagnosis. There was a direct and indirect bidirectional effect through the glial fibrillary acidic protein of WMH on phosphorylated tau 217 concentration, which was associated with neurofilament light chain concentration in the entire sample. Amongst cognitively stable participants, WMH was directly and indirectly, through glial fibrillary acidic protein, associated with phosphorylated tau 217 concentration, and in those with mild cognitive impairment, there was a direct effect of WMH on phosphorylated tau 217 and neurofilament light chain concentrations. There were no associations of WMH with biomarker concentrations among those diagnosed with dementia. The findings from this cross-sectional study suggest that among individuals with Down syndrome, cerebrovascular disease promotes neurodegeneration by increasing astrocytosis and tau pathophysiology in the presymptomatic phases of Alzheimer’s disease, but future studies will need to confirm these associations with longitudinal data. This work joins an emerging literature that implicates cerebrovascular disease and its interface with neuroinflammation as a core pathological feature of Alzheimer’s disease in adults with Down syndrome.

## Teaser

Edwards *et al*. report an association of an MRI marker of cerebrovascular disease with plasma markers of phosphorylated tau protein and neurodegeneration that appears to be mediated by astrocytosis in the presymptomatic phases of Alzheimer’s disease among adults with Down syndrome.

## Graphical Abstract

**Graphical abstract:**
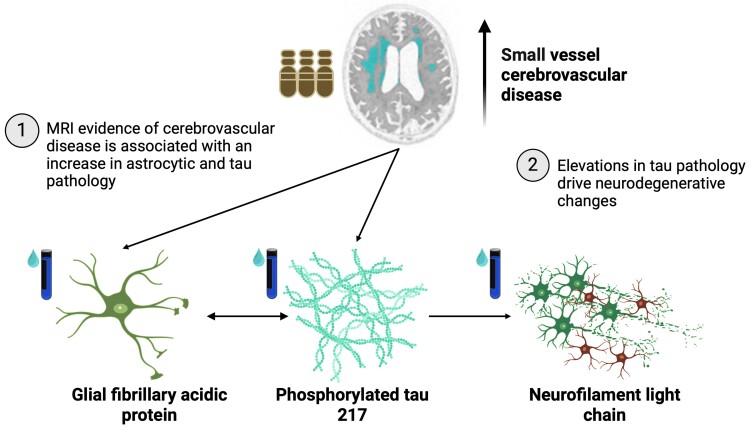

## Introduction

Virtually all individuals with Down syndrome develop Alzheimer’s disease pathology, including abnormal amyloid-beta (Aβ) plaques and tau neurofibrillary tangles, by the age of 40 years,^1,2^ and most develop dementia by the age of 60.^3^ Down syndrome is considered a genetic form of Alzheimer’s disease,^4^ and pathogenesis is attributable to the triplication of chromosome 21, which contains the amyloid precursor protein (APP) coding gene.^5^ Models of Alzheimer’s disease progression in both late-onset and genetic forms emphasize the role of Aβ in initiating tau pathology and subsequent neurodegeneration, sometimes referred to as the ‘ATN framework’.^6^ While there is general support for this pathophysiological cascade,^6,7^ there is increasing evidence that additional pathways may promote Alzheimer’s disease pathogenesis and progression.^8^

Cerebrovascular disease contributes to the risk and course of clinical Alzheimer’s disease and increases the likelihood of developing dementia.^9,10^ Neuroimaging biomarkers for small vessel cerebrovascular disease, including white matter hyperintensities (WMH), are associated with neurodegeneration, indexed by Alzheimer’s disease-related patterns of cortical atrophy and fluid biomarker concentrations.^11-13^ Despite consistent observations of its occurrence and contributions to clinical outcomes in people with Alzheimer’s disease, cerebrovascular disease is generally considered a common comorbidity with Alzheimer’s disease that is not a hallmark characteristic.^6^

Populations at genetic risk provide insight into the extent to which cerebrovascular disease represents a ‘core feature’ of Alzheimer’s disease. Among community-dwelling older adults, those carrying the *APOE* ɛ4 allele, the strongest genetic risk factor for late-onset Alzheimer’s disease, have greater degrees of cerebrovascular disease than non-ɛ4 carriers.^14^ Despite their younger age and relatively low vascular risk factor profiles, individuals with autosomal dominant, fully penetrant mutations for Alzheimer’s disease have increased WMH volumes up to 20 years prior to expected symptom onset compared with individuals without genetic mutations for Alzheimer’s disease but who are at similar risk for inheriting the mutation.^13^ Such changes account for more variance in cognition than do other Alzheimer’s disease biomarkers.^15^ Similarly, individuals with Down syndrome generally have lower degrees of vascular risk compared with neurotypical adults and seem to be protected against developing hypertension,^16-18^ yet there is neuroimaging evidence of cerebrovascular disease that increases with the clinical progression of Alzheimer’s disease^19^ that we hypothesize is mediated by inflammation and/or upstream genetic factors.

Evidence from late-onset and genetic forms of Alzheimer’s disease suggests that cerebrovascular pathology is indeed a prominent feature of Alzheimer’s disease that cannot be attributable solely to exposure to vascular risk factors, but whether cerebrovascular disease promotes primary Alzheimer’s disease pathophysiological progression remains unclear. Reports of associations between cerebrovascular disease and Alzheimer’s disease biomarkers are mixed, with some showing codependency^20^ and others not.^21,22^ In an animal model of WMH, we found that white matter damage induced by transient hypoperfusion promotes tau hyperphosphorylation, but it is unclear what factors mediate this effect.^12^

Emerging work suggests the critical role of neuroinflammation, mainly manifesting as a change in microglia morphology,^23-26^ astrocytosis^27-29^ and inflammatory mediators,^30^ in Alzheimer’s disease pathogenesis and course, with emerging evidence of intimate crosstalk between inflammatory processes and the brain’s vasculature.^31^ In adults with Down syndrome, MRI markers of cerebrovascular disease are associated with proteomic patterns reflective of inflammation earlier in the disease and with patterns reflective of neurodegeneration later in the disease.^32^ Glial fibrillary acidic protein (GFAP) is a cytoskeletal protein found in astrocytes, released during astrogliosis, and can be measured reliably in cerebrospinal and blood compartments as a surrogate measure of astrocytosis.^33-36^ Plasma and cerebrospinal fluid (CSF) GFAP concentration is elevated in people with and at risk for Alzheimer’s disease^37-39^ and appears to mediate the relationship between Aβ and tau pathology.^40,41^ In adults with Down syndrome, plasma GFAP concentration discriminates between individuals who are asymptomatic and those diagnosed with Alzheimer’s disease.^42^ Further, GFAP levels are strongly correlated with indicators of Aβ and tau pathology, neurodegeneration and clinical progression of Alzheimer’s disease in adults with Down syndrome.^42-44^

In the current study, we examined the association between WMH, as a marker of small vessel cerebrovascular disease, and Alzheimer’s disease plasma biomarker concentrations, including Aβ40/Aβ42, phosphorylated tau 217 (p-tau217) and GFAP, with neurofilament light chain (NfL) across disease stages in adults with Down syndrome. Because astrocytosis (i) is prominent around blood vessels in Alzheimer’s disease^45^; (ii) induced cerebral hypoperfusion, a characteristic of Down syndrome^46^; increases the number of GFAP-positive astrocytes^47^; and (iii) is an early disease feature of Alzheimer’s disease,^48^ we used a series of mediation and path analyses applied to cross-sectional data to test our hypothesis that cerebrovascular disease gives rise to tau pathology and ultimately neurodegeneration via astrocytosis across different Alzheimer’s disease stages in adults with Down syndrome.

## Materials and methods

### Participants and participant diagnosis

Participants came from the Alzheimer’s Biomarkers Consortium–Down Syndrome (ABC-DS), a multisite, observational study designed to examine biomarker, clinical and genetic correlates of and contributors to Alzheimer’s disease among adults with Down syndrome.^49^ The sample included 185 individuals with trisomy 21 from the Neurodegeneration in Aging Down Syndrome (NiAD; U01 AG051406) and Biomarkers of Alzheimer’s Disease in Adults with Down Syndrome (ADDS; U01 AG051412), both of which are now contained within ABC-DS. For the current study, participants with available MRI data and derived plasma biomarkers of interest were selected for analysis. One hundred thirty-eight participants characterized as cognitively stable, 24 patients with mild cognitive impairment (MCI), 16 patients with Alzheimer’s disease dementia (Down syndrome–Alzheimer’s disease) and 8 with diagnoses that were ‘unable to be determined’ were included. Diagnoses were based on a consensus conference that reviewed available neuropsychological and clinical data, as described previously in detail.^49^ In short, clinical experts in the assessment and diagnosis of Alzheimer’s disease in Down syndrome performed a standardized clinical evaluation of each participant, which considered functional abilities and health history. Participants were then assigned one of four Alzheimer’s disease-related diagnoses (i.e. cognitively stable, MCI, Down syndrome–Alzheimer’s disease, unable to be determined).

#### Magnetic resonance imaging

MRI scans were acquired at ADDS and NiAD participating sites. NiAD sites acquired 2D T2-weighted fluid-attenuated inversion recovery (FLAIR) scan [repetition time (TR)/echo time (TE)/inversion time (TI) = 5000/386/1800 ms, voxel size = 0.4 × 0.4 × 0.9mm^3^] and ADDS sites acquired 3D T2-weighted FLAIR scan (TR/TE/TI = 4800/119/1473 ms, voxel size = 0.9 × 0.9 × 0.5mm^3^).

White matter hyperintensity volume was quantitated with in-house software. Briefly, FLAIR images were reconstructed to a uniform matrix of 256 × 256 × 256 with a voxel size of 1 mm^3^. The images were reoriented to standard anatomical space (MNI152), skull stripped and bias field corrected.^50,51^ The images were processed through a customized module designed to extract percentile thresholds from the intensity histogram of each image automatically.^52,53^ Next, a white matter segment was created using the convolutional neural networks tool.^54^ Two specific percentile thresholds were computed: one for the transition between dark and bright voxel intensity and another for the transition between bright and brightest voxel intensity. These thresholds initialized a Gaussian mixture model (GMM) and expectation–maximization algorithm^55^ within the white matter segment of the FLAIR images, using two components to represent hyperintense and non-hyperintense voxels.

Following the computation of percentile thresholds, we calculated the inter-percentile range (IPR) between these values and introduced a relaxed threshold of 10 to account for variations in FLAIR image quality. This adjustment was made by applying a multiplicative factor to the IPR.

**Figure 1:**
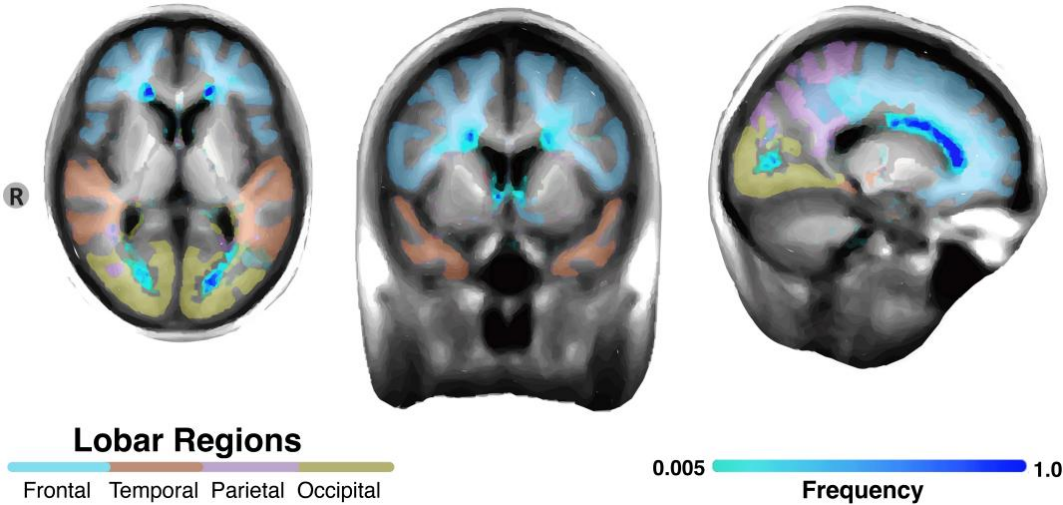
**Frequency map of white matter hyperintensities in adults with Down syndrome.** A voxel-wise frequency map of WMH was created by summing voxels labelled across all 185 individual 3D and dividing by 185. Each voxel’s value represents the proportion of times it was labelled as a WMH across the 185 masks from low frequency (light blue) to high frequency (dark blue).

Finally, probability distribution maps were generated to represent the segmented WMH within the FLAIR images. The Roberts edge detection function^56^ was applied to the probability distribution maps, ensuring the removal of any non-white matter voxels from the brain’s contour. The labelled voxels were added together and multiplied by voxel dimensions to calculate the total WMH volume in cubic centimetre. Figure 1 displays the voxel-wise frequencies of WMH across all participants.

##### Plasma samples and analysis

Plasma Aβ42, Aβ40, p-tau217, NfL and GFAP concentrations were derived for each participant from plasma samples as previously described.^43^ Plasma samples were shipped to the University of North Texas where Aβ42, Aβ40 and NfL concentrations were quantified with single molecule array (Simoa) assays (Quanterix). We calculated the ratio of Aβ42 to Aβ40 as the biomarker for amyloid pathology.^57^ Plasma samples from the same group of participants were shipped to Lund University for quantification of p-tau217 and GFAP concentrations. The p-tau217 concentration was assayed according to the published protocols using immunoassay on a Meso Scale Discovery platform developed by Lilly Research Laboratories as previously described.^44,58^ GFAP concentration was quantified using Simoa assays (Quanterix). We calculated age-residualized values for NfL concentration values, our primary dependent variable, because the pathophysiological progression of Alzheimer’s disease among individuals with Down syndrome is strongly age-dependent.^1,2^ This age dependency may induce epiphenomenological relationships among Alzheimer’s disease-related variables when conducting cross-sectional analyses due to their shared association with age. On the other hand, statistical adjustment for age may obscure important associations among factors whose variance is strongly age-dependent. Therefore, we chose to operationalize neurodegeneration as age-residualized NfL in the subsequent analyses.

##### Statistical analysis

###### Association of biomarkers with neurodegeneration

We examined the association of WMH and each Alzheimer’s-related biomarker concentration with age-residualized NfL in the entire sample and stratified by diagnosis with bivariate Pearson correlations. Participants with an undetermined diagnosis were not included in any analyses stratified by diagnosis.

###### Mediation analyses

We conducted a series of causal mediation analyses in the entire sample informed by the observed bivariate associations. We used the ‘mediation’ package in R^59^ to examine whether (i) GFAP mediates the relationship between WMH volume and p-tau217 concentration; (ii) whether p-tau217 concentration mediates the relationship between WMH volume and NfL concentration; and (iii) whether p-tau217 concentration mediates the relationship between GFAP and NfL concentrations. To probe directionality, we ran models in which the hypothesized predictor and mediator were switched. The average causal mediation effect (ACME), the portion of the direct effect on the outcome that is attributable to the mediator’s effect and the corresponding *P*-value were extracted from each mediation model.

###### Path analysis

We tested our *a priori* hypothesis of pathophysiological cascade that is initiated by cerebrovascular disease with a path analysis in the combined sample and in groups stratified by Alzheimer’s disease-related diagnosis. Notably, path analysis, while still based on principles of correlation, can be used to test alternative models to understand the most likely direction and possible causal relationships among cross-sectional data.^60^ For stratified analyses, we removed the participants with clinical diagnoses that were unable to be determined; however, data from these participants were included in the analyses with the combined sample, which did not consider diagnosis explicitly. The path analysis tested the effect of WMH on downstream neurodegeneration (i.e. age-residualized NfL concentrations) via GFAP and p-tau217. The paths were estimated using the ‘lavaan’ package in R,^61^ which established the direct and indirect effects among biomarkers, and the model was fit using the sem() function. All analyses were adjusted for research site. In a *post hoc* analysis, we examined a model in which age was formally examined as a driver of pathological accumulation.

## Results

Sample characteristics across diagnostic groups are reported in Table 1. Cognitively stable participants and those with an undetermined diagnostic status were younger than those with MCI and Down syndrome–Alzheimer’s disease, and a smaller proportion of women was diagnosed with MCI than other groups. There were no differences in reported history of hypertension, hypotension, Type 1 or 2 diabetes or hypercholesterolaemia (Table 1).

**Table 1:** Sample characteristics by diagnostic group

	Cognitively stable	MCI	Down syndrome–Alzheimer’s disease	Undetermined	Whole sample	Test statistic	*P*-value
***n***	137	24	16	8	185		
***Demographic***							
*Age, mean (SD) years*	43 (9.1)	51 (5.8)	54.5 (6)	48.4 (8.8)	45.2 (9.3)	*F* = 27.05	<0.001
*Women, n (%)*	64 (47)	4 (17)	9 (56)	4 (50)	81 (44)	*χ* ^2^ = 8.7	0.003
***Vascular risk factors***							
*Hypertension, n (%)*	3 (2)	0 (0)	0 (0)	0 (0)	3 (2)	χ^2^ = 4.1	0.26
*Hypotension, n (%)*	3 (2)	1 (4)	0 (0)	0 (0)	4 (2)	*χ* ^2^ = 3.5	0.32
*Type 1 diabetes, n (%)*	0 (0)	0 (0)	0 (0)	0 (0)	0 (0)		
*Type 2 diabetes, n (%)*	2 (2)	0 (0)	0 (0)	0 (0)	2 (1)	*χ* ^2^ = 0.9	0.82
*Hypercholesterolaemia, n (%)*	15 (11)	1 (4)	2 (13)	1 (13)	19 (1)	*χ* ^2^ = 1.1	0.3

MCI, mild cognitive impairment.

We confirmed strong associations between age and Alzheimer’s disease biomarker concentrations, apart from Aβ42/40: GFAP, *r* = 0.612 (0.513, 0.695), *P* < 0.0001; NfL, *r* = 0.523 (0.409, 0.62), *P* < 0.0001; *P*-tau217, *r* = 0.379 (0.248, 0.496), *P* < 0.0001; Aβ42/40, *r* = 0.107 (−0.036, 0.248), *P* = 0.147.

### Associations of plasma biomarkers and WMH with age-residualized NfL concentration across diagnostic groups

Table 2 displays the associations of plasma biomarkers and WMH volume with age-residualized NfL concentration. White matter hyperintensity volume, GFAP concentration and p-tau217 concentration were positively associated with age-residualized NfL in the entire sample. In cognitively stable participants, neither WMH volume nor plasma Alzheimer’s disease biomarker concentrations were associated with age-residualized NfL levels, likely reflecting the limited amount of variance in these factors at this disease stage. Among those with MCI, increased WMH volume was associated with higher age-residualized NfL, while increased GFAP concentration and p-tau217 concentration were associated with higher age-residualized NfL in participants with MCI and Alzheimer’s disease. White matter hyperintensity volume was not associated with higher age-residualized NfL in participants with Alzheimer’s disease.

**Table 2:** Associations of plasma biomarkers and WMH with age-residualized NfL concentrations across diagnostic groups

	Whole sample		Cognitively stable		MCI		Down syndrome–Alzheimer’s disease		Undetermined	
	*r* (CI)	*P*-value	*r* (CI)	*P*-value	*r* (CI)	*P*-value	*r* (CI)	*P*-value	*r* (CI)	*P*-value
WMH volume	0.32 (0.18, 0.44)	<0.0001	0.14 (−0.03, 0.30)	0.10	0.62 (0.23, 0.80)	0.003	0.42 (0.18, 0.85)	0.12	0.44 (−0.39, 0.87)	0.32
Aβ42/40	0.14 (−0.01, 0.28)	0.06	0.12 (−0.05, 0.28)	0.18	−0.14 (−0.52, 0.28)	0.51	0.31 (−0.22, 0.70)	0.24	−0.41 (−0.87, 0.41)	0.31
p-tau217	0.52 (0.41, 0.62)	<0.0001	0.10 (−0.07, 0.26)	0.25	0.86 (0.70, 0.94)	<0.0001	0.82 (0.55, 0.94)	<0.0001	0.21 (−0.58, 0.80)	0.61
GFAP	0.31 (0.18, 0.44)	<0.0001	0.09 (−0.081, 0.25)	0.31	0.47 (0.08, 0.74)	0.02	0.62 (0.25, 0.87)	0.005	0.19 (−0.59, 0.79)	0.65

Pearson correlation tests examine the associations of plasma biomarkers and WMH volume (cubic centimetre) with age-residualized NfL concentrations across diagnostic groups.

Aβ, amyloid beta; WMH, white matter hyperintensities; p-tau217, phosphorylated tau 217; GFAP, glial fibrillary acidic protein; NfL, neurofilament light chain; MCI, mild cognitive impairment.

#### Mediation analyses

**Figure 2:**
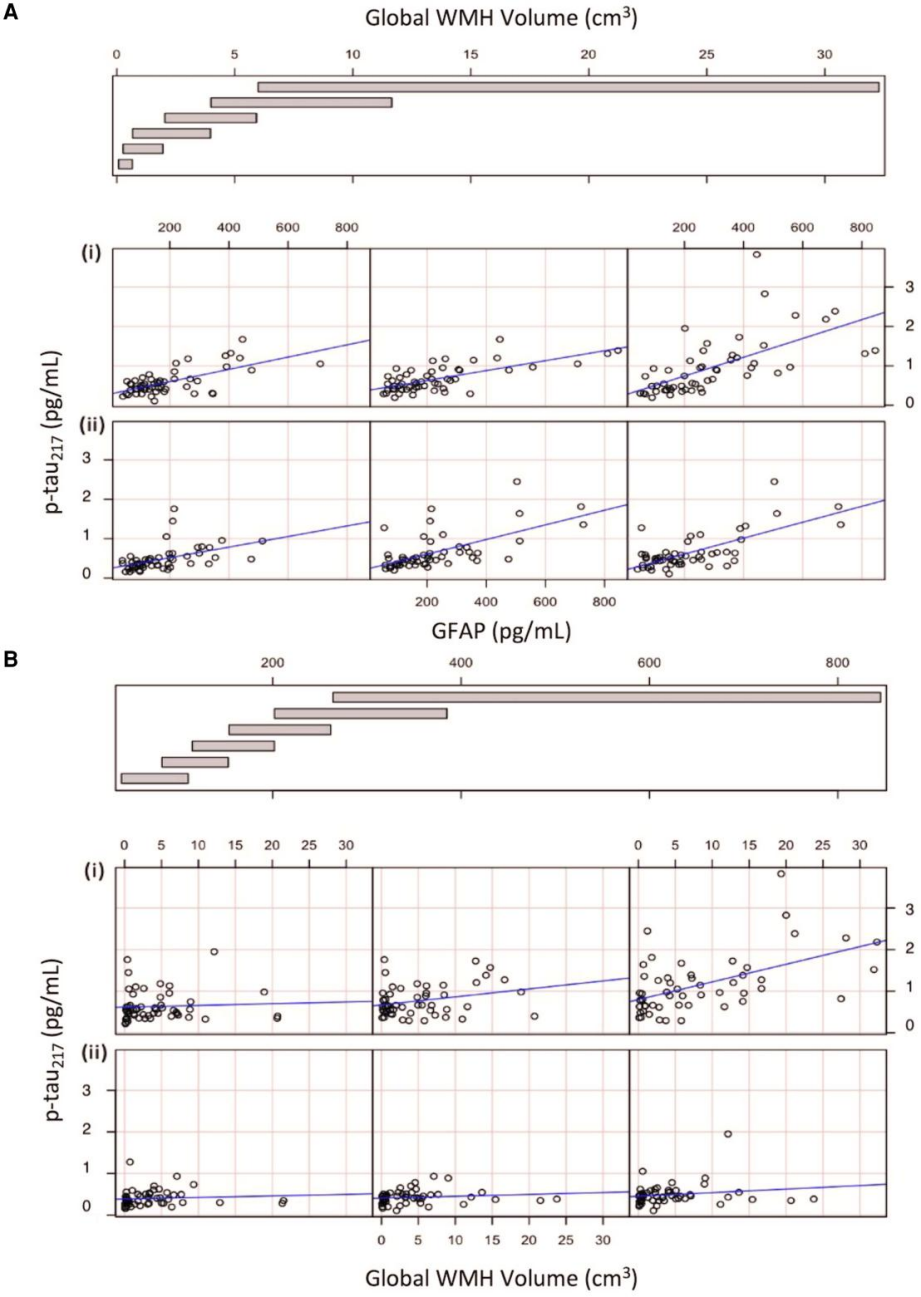
**Conditional relationship between WMH and GFAP on p-tau217 concentration.** Relationship between GFAP and p-tau217 concentration conditioned by WMH volume (**A**)and relationship between WMH and p-tau217 concentration conditioned by GFAP (**B**). The plots show the relationship between GFAP or WMH and p-tau217 for different ranges of WMH and GFAP, respectively. The panels are read from bottom left to top right along each row with the bottom row representing the lowest range of WMH volume or GFAP concentration and the top row representing the highest range of WMH volume or GFAP concentration, respectively. The rows demonstrating the relationship in individuals with higher distributions are indicated by rows labelled (ii) while relationships in participants with lower distributions are indicated by rows labelled (i). The columns correspond to the levels of WMH or GFAP as shown in the bar graph above the panels. For example, in Fig. 2A, the top right plot shows the relationship between GFAP and p-tau217 in individuals with the largest WMH volume (i) while the bottom left panel shows the relationship between GFAP and p-tau217 in individuals with the smallest WMH volume (ii). WMH, white matter hyperintensities; GFAP, glial fibrillary acidic protein; p-tau217, phosphorylated tau 217.

Three results emerged from the statistical mediation analyses. First, p-tau217 concentration mediated the relationship between WMH and age-residualized NfL concentration {ACME [confidence interval (CI)] = 0.44 (0.17, 0.83}, *P* < 0.0001]. When we reversed the independent variable and mediator variable and re-ran the analyses, WMH did not mediate an association between p-tau217 concentration and NfL [ACME (CI) = 0.88 (−0.66, 2.50), *P* = 0.22]. Second, GFAP concentration mediated the relationship between WMH and p-tau217 concentration [ACME (CI) = 0.0201 (0.01, 0.02), *P* < 0.001]; the reverse model revealed a congruent mediation effect of WMH, albeit to a lesser extent, on the relationship between GFAP and p-tau217 [ACME (CI) = 0.0003 (0.0001, 0.0002), *P* < 0.001]. Third, p-tau217 concentration mediated the relationship between GFAP concentration and age-residualized NfL concentration [ACME (CI) = 0.04 (0.01, 0.05), *P* < 0.0001]. When reversed, GFAP did not mediate an association between p-tau217 concentration and NfL [ACME (CI) = −0.80 (−2.96, 2.66), *P* = 0.67]. A *post hoc* analysis revealed an interaction between WMH and GFAP on p-tau217 concentration, such that WMH was most strongly associated with p-tau217 in the presence of elevated GFAP while GFAP was most strongly associated with p-tau217 in individuals with high WMH volume (see Fig. 2).

##### Path analysis

**Figure 3:**
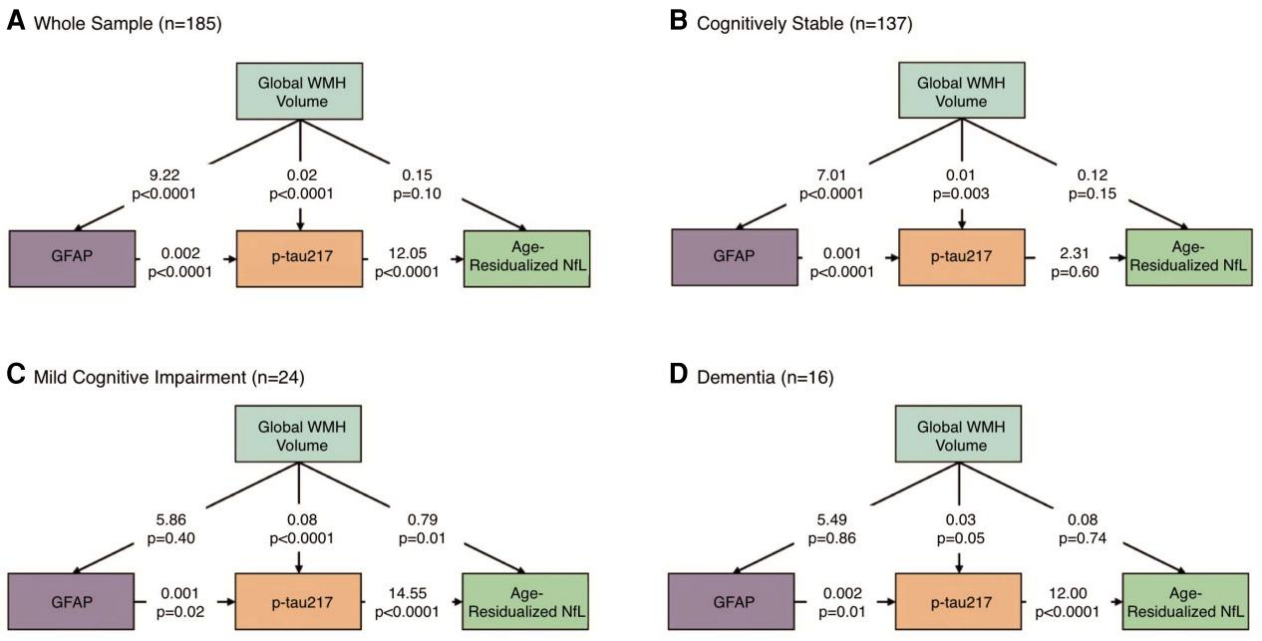
**Path models for biomarker progression across diagnostic groups.** Structural equation modelling calculates relative causal relationships among different pathophysiological contributors in the whole sample (**A**) and across diagnostic groups (**B–D**). Larger numbers (regression coefficients) signify stronger direct effects. Aβ, amyloid beta; WMH, white matter hyperintensities; p-tau217, phosphorylated tau 217; GFAP: glial fibrillary acidic protein; NfL, neurofilament light chain; MCI, mild cognitive impairment.

Informed by the relationships observed in the mediation analyses, we conducted a path analysis to examine statistical causality within our hypothesized pathophysiological cascade in the entire sample and stratified by diagnosis. In the entire sample (Fig. 3A), the analysis revealed a cascade initiated by WMH, which had a direct and an indirect effect through GFAP on p-tau217 concentration. P-tau217 concentration, in turn, was associated with age-residualized NfL concentration. In this combined sample, increasing p-tau217 concentration was primarily attributable to increasing WMH volume, while increases in NfL were mainly related to increasing p-tau217 concentration. Among cognitively stable participants (Fig. 3B), there was a direct and indirect effect through GFAP of WMH on p-tau217 concentration, but p-tau217 concentration was not associated with NfL concentration. No Alzheimer’s disease biomarker concentrations were associated with age-residualized NfL concentrations among cognitively stable participants, likely due to low variance in neurodegeneration at this disease stage. Among those with MCI (Fig. 3C), increased WMH volume had a direct effect on p-tau217 and NfL concentrations but not GFAP concentration. GFAP concentration had an indirect effect on NfL concentration through p-tau217. Finally, in those diagnosed with dementia (Fig. 3D), there were no direct or indirect effects of WMH on plasma Alzheimer’s disease biomarker concentrations. Still, GFAP continued to have a positive indirect effect on NfL through p-tau217 concentration. In a path analysis that formally included age, age drove increases in WMH, which in turn were associated with elevations in plasma GFAP, p-tau217 and NfL concentrations (Supplementary Fig. 1).

## Discussion

Our findings suggest that among adults with Down syndrome, cerebrovascular disease promotes Alzheimer’s disease-related neurodegeneration indirectly through increasing astrocytosis and tau pathophysiology in the preclinical stages of Alzheimer’s disease and directly and indirectly in the clinical stages of Alzheimer’s disease. These results support our hypothesis that cerebrovascular disease in the form of WMH may initially promote increases in inflammation and tau pathophysiology, giving rise to downstream neurodegeneration.

Pathogenic models of Alzheimer’s disease emphasize a precipitating role of Aβ that leads to tau pathology and subsequent neurodegeneration^6^; however, we found that tau pathology did not appear to exert a direct effect on neurodegeneration until elevated by both cerebrovascular disease and astrocytosis. *Post hoc* analyses revealed an interaction between plasma GFAP and WMH in promoting Alzheimer’s disease pathophysiology and downstream neurodegeneration, suggesting synergy between vascular and inflammatory processes in this proposed pathophysiological cascade.

Our findings are in line with our previous study, which showed more consistent associations between peripheral proteomic markers of inflammation and MRI markers of cerebrovascular disease in presymptomatic phases of Alzheimer’s disease among adults with Down syndrome.^32^ Further, post-mortem data revealed a unique inflammatory profile in adults with Down syndrome and inflammatory proteins related to astrocytosis were elevated in the early stages of Alzheimer’s disease.^62^ Adults with Down syndrome also show evidence of blood–brain barrier (BBB) disruption at autopsy,^63^ which may be the result, in part, of vascular lesions observed on MRI.^64^ Additionally, in mouse models of Alzheimer’s disease, white matter abnormalities caused by hypoperfusion promote Alzheimer’s disease pathology,^12^ astrocytosis^65^ and BBB disruption.^66^ Therefore, it is possible that cerebrovascular lesions give rise to astrocytosis and BBB disruption, which promote downstream tau accumulation and neurodegeneration.

White matter hyperintensities are generally considered to reflect ‘end-organ’ ischaemic damage due to chronic exposure to vascular risk factors.^67^ In the context of Alzheimer’s disease, however, the aetiology of WMH has been widely debated.^67^ Some argue that in Alzheimer’s disease, WMH are attributable primarily to cerebral amyloid angiopathy (CAA),^68^ especially in the absence of systemic vascular risk factors, as in Down syndrome. Others claim that WMH are the *result* of Alzheimer’s disease-related neurodegeneration, so-called Wallerian degeneration.^69,70^ We have argued against some of these pathways on the basis of temporality, experimental evidence and anatomical distribution,^71^ and the results of this study provide further evidence against these possibilities. In *post hoc* analyses, we did not observe an association between WMH volume and number of cerebral microbleeds [*r* = −0.043 (−0.226, 0.143), *P* = 0.649], a radiological marker of CAA.^19,72^ This observation is consistent with our finding that cerebral microbleeds only modestly mediate an association between autosomal dominant mutations for Alzheimer’s disease and increased WMH volume.^73^ While new criteria for CAA include multispot subcortical WMH,^74^ this pattern of WMH is rarely observed in individuals with Down syndrome upon visual inspection. Instead, WMH tend to be confluent with the walls of the lateral ventricles and appear as diffuse lesions extending towards the cortex as they progress. Further, the emergence of WMH and amyloid occurs at approximately the same age in adults with Down syndrome.^75^ It is unlikely that WMH are solely attributable (downstream) to amyloidosis in this population. Instead, we hypothesize that WMH in the context of Alzheimer’s disease (and in the relative absence of vascular risk factors) reflect inflammatory changes at the level of the endothelium, which could contribute to the weakening of the vessel walls and increased risk for other vascular lesions, like microbleeds, as well as the downstream tau abnormalities we observed. Nonetheless, WMH may be caused by multiple pathologies, and clearly future work should attempt to clarify their aetiological and pathological basis in individuals with and without Alzheimer’s disease.

Our statistical modelling suggests that WMH precede or are upstream from tau pathophysiology and neurodegeneration markers. Animal stroke models show evidence of increased GFAP-positive astrocytes observed around the lesion days after vessel occlusion in models of small vessel and white matter stroke^76,77^ and white matter hypoperfusion increases tau hyperphosphorylation in mouse models.^12^ Taken together, we speculate that there is an endogenous cerebrovascular component to Alzheimer’s disease pathogenesis that likely is not due to amyloid and tau pathology but instead interacts with inflammatory processes to promote tauopathy and subsequent neurodegeneration.

It is unclear what upstream factors among individuals with Down syndrome give rise to WMH and other cerebrovascular lesions observed on MRI in the absence of vascular risk factors. Individuals with Down syndrome have a unique inflammatory profile and elevated reactive oxygen species (ROS) reflecting mitochondrial dysfunction,^78^ which could potentially cause vascular inflammation and reflect BBB dysfunction.^79,80^ ROS are also increased in mouse models of hypoperfusion-induced oxidative stress, which interferes with white matter repair.^81-83^ This profile is likely mediated by the antioxidant enzyme Cu/Zn-superoxide dismutase-1 (*SOD1*) gene localized on chromosome 21.^84,85^ The oligodendrocyte lineage transcription factor 2 (*OLIG2*) gene is also located on chromosome 21.^86^*OLIG2* expresses in oligodendrocyte progenitor cells (OPCs), which regulate white matter development,^87^ myelin maintenance and myelin repair, features that may be central to Alzheimer’s disease.^88-90^ As the WMH observed on MRI may partially reflect demyelination and axonal damage,^91^ the downstream products of *OLIG2* overexpression in Down syndrome and its interaction with inflammatory processes may be in the pathway towards the development of cerebrovascular lesions in the white matter.^92^ Triplication of *OLIG2* results in an increased number of OPCs, shifting differentiation towards an increased number of astrocytes in Down syndrome^92^ that may serve to impede OPC-mediated remyelination and repair, possibly^93^ contributing to the strong relationship between cerebrovascular disease and astrocytosis reported here. Future research should examine potential pathways that give rise to cerebrovascular disease in Down syndrome and determine the extent to which they are independent or interact with Alzheimer’s disease pathophysiology.

Notably, we did not find any association between plasma Aβ42/40 concentration and WMH volume or NfL concentration. This finding was unexpected, given the well-documented overproduction of Aβ in individuals with Down syndrome.^1^ However, plasma Aβ concentrations remain steady across the adult lifespan in adults with Down syndrome after about age 30 years^94^ and may have plateaued in most participants prior to enrolment. The lack of dynamic range in Aβ concentrations could yield null results despite the importance of the amyloid pathology. Additionally, plasma amyloid measures may not capture brain-related amyloid pathology with as high fidelity as the other plasma Alzheimer’s disease biomarkers.^95,96^ On the other hand, while p-tau217 reliably reflects tau-positron emission tomography (PET) and CSF levels,^97,98^ increases in p-tau217 concentration precede elevations in tau-PET^99^ and capture some degree of Aβ pathology.^98,100^ However, in adults with Down syndrome, p-tau217 is more strongly associated with tau-PET standard uptake value ratio (SUVR) than Aβ-PET (Centiloid) Aβ+ individuals,^44^ and in *post hoc* analyses among participants in ABC-DS analyses, we found that p-tau217 was more strongly associated with Braak I–II [*R* = 0.66 (0.55, 0.74), *P* < 0.0001] and Braak III–IV [*R* = 0.75 (0.67, 0.82), *P* < 0.0001] tau-PET SUVR than with Aβ Centiloid value [*R* = 0.64 (0.55, 0.72), *P* < 0.0001], suggesting that p-tau217 concentrations in the current study reflect primarily tau pathology.

There are some limitations to this study. Although we used statistical analyses that probe directionality, the data used in the study were cross-sectional, which limits our ability to confirm causal relationships. Longitudinal data will be helpful to confirm the temporal relationships among cerebrovascular disease, astrocytosis, tau pathophysiology and neurodegeneration fully. Further, there were relatively small sample sizes for subgroups of participants, including those diagnosed with MCI and dementia. However, the relationships we observed within these groups were statistically reliable, and we therefore had adequate statistical power for hypothesis testing. Finally, the development, implementation and understanding of fluidic Alzheimer’s disease-related biomarkers are rapidly evolving, and the underlying factors that drive variance in these measures are not fully understood. Future work will combine these observations with other data sources and cohorts, including molecular PET imaging and pathological outcomes, to further the understanding of the role of cerebrovascular disease in Alzheimer’s disease among individuals with Down syndrome and neurotypical adults. Nonetheless, our study provides evidence suggesting that cerebrovascular disease and inflammation play a key role early in Alzheimer’s disease-related neurodegeneration in adults with Down syndrome.

## Supplementary Material

fcae331_Supplementary_Data

## Data Availability

Qualified investigators can submit requests for access to data and samples from ABC-DS (https://pitt.co1.qualtrics.com/jfe/form/SV_cu0pNCZZlrdSxUN), and all requests will be reviewed by ABC-DS investigators and NIH staff.

## Appendix

### Alzheimer’s Biomarkers Consortium–Down Syndrome investigators

Howard J. Aizenstein, Beau M. Ances, Howard F. Andrews, Karen Bell, Rasmus M. Birn, Adam M. Brickman, Peter Bulova, Amrita Cheema, Kewei Chen, Bradley T. Christian, PhD; Isabel Clare, Ann D. Cohen, John N. Constantino, Eric W. Doran, Natalie C. Edwards, Anne Fagan, Eleanor Feingold, Tatiana M. Foroud, Benjamin L. Handen, Jordan Harp, Sigan L. Hartley, Elizabeth Head, Rachel Henson, Christy Hom, Lawrence Honig, Milos D. Ikonomovic, Sterling C Johnson, Courtney Jordan, M. Ilyas Kamboh, David Keator, William E. Klunk, Julia K. Kofler, William Charles Kreisl, Sharon J. Krinsky-McHale, Florence Lai, MD; Patrick Lao, Charles Laymon, Joseph H. Lee, Ira T. Lott, Victoria Lupson, Mark Mapstone, Chester A. Mathis, Davneet Singh Minhas, Neelesh Nadkarni, Sid O’Bryant, Melissa Parisi, Deborah Pang, Melissa Petersen, Julie C. Price, Margaret Pulsifer, Michael S. Rafii, Eric Reiman, Batool Rizvi, Herminia Diana Rosas, Laurie Ryan, Frederick Schmitt, Nicole Schupf, Wayne P. Silverman, Dana L. Tudorascu, Rameshwari Tumuluru, Benjamin Tycko, Badri Varadarajan, Desiree A. White, Michael A. Yassa, Shahid Zaman, Fan Zhang.
