# Supplementary material for: Cerebrovascular disease is associated with Alzheimer’s plasma biomarker concentrations in adults with Down syndrome
Source: Brain Commun. 2024 Sep 25;6(5):fcae331. doi: 10.1093/braincomms/fcae331 (PMC11472828; doi:10.1093/braincomms/fcae331)
Supplement: fcae331_Supplementary_Data [file fcae331_supplementary_data.zip › Supplementary_Figure_1_Legend.docx]

**Supplementary Figure 1. Path models for age-driven biomarker progression.**

Structural equation modeling calculates relative causal relationships among different pathophysiological contributors. Larger numbers (regression coefficients) signify stronger direct effects.

*WMH: white matter hyperintensities, p-tau217: phosphorylated tau 217, GFAP: glial fibrillary acidic protein, NfL: neurofilament light chain.*
