# Supplementary material for: Cerebrovascular disease is associated with Alzheimer’s plasma biomarker concentrations in adults with Down syndrome
Source: Brain Commun. 2024 Sep 25;6(5):fcae331. doi: 10.1093/braincomms/fcae331 (PMC11472828; doi:10.1093/braincomms/fcae331)
Supplement: fcae331_Supplementary_Data [file fcae331_supplementary_data.zip › Supplementary_Material_Analysis_Script.docx]

**Supplementary Material: Analysis Script**

########### Load Packages ###########

install.packages("lm.beta")

library("lm.beta")

library(nlme)

library(ggplot2)

library(qplot)

source('http://openmx.psyc.virginia.edu/getOpenMx.R')

install.packages("lavaan")

library(lavaan)

install.packages("semPlot")

library(semPlot)

install.packages("OpenMx")

library(OpenMx)

install.packages("tidyverse")

library(tidyverse)

install.packages("knitr")

library(knitr)

install.packages("kableExtra")

library(kableExtra)

install.packages("GGally")

library(GGally)

install.packages("mediation")

library(mediation)

install.packages("mtvnorm")

library(mvtnorm)

install.packages("Hmisc")

library(Hmisc)

install.packages("lattice")

library(lattice)

install.packages("tidySEM")

library(tidySEM)

if(!require("ppcor")){

install.packages("ppcor", repos='http://cran.us.r-project.org')

library(ppcor)

}

########### Load in data sets ###########

master <- read.csv("")

master_CS <- read.csv("")

master_MCI <- read.csv("")

master_AD <- read.csv("")

master_UD <- read.csv("")

########### Correlations ###########

cor.test(master$age,master$WMH)

cor.test(master$age,master$ab4240)

cor.test(master$age,master$ptau217)

cor.test(master$age,master$NfL)

cor.test(master$age,master$GFAP)

########### Age regression correlations (Table 2) ###########

# NfL

lm1 <- lm(NfL~ age, data = master)

lm1.res = resid(lm1)

lm2 <- lm(NfL~ age, data = master_CS)

lm2.res = resid(lm12)

lm3 <- lm(NfL~ age, data = master_MCI)

lm3.res = resid(lm13)

lm4 <- lm(NfL~ age, data = master_AD)

lm4.res = resid(lm14)

lm5 <- lm(NfL~ age, data = master_UD)

lm5.res = resid(lm5)

cor.test(master$WMH,lm11.res, method = "pearson")

cor.test(master_CS$WMH,lm2.res, method = "pearson")

cor.test(master_MCI$WMH,lm3.res, method = "pearson")

cor.test(master_AD$WMH,lm1.res, method = "pearson")

cor.test(master_UD$WMH,lm5.res, method = "pearson")

cor.test(master$ab4240,lm1.res, method = "pearson")

cor.test(master_CS$ab4240,,lm2.res, method = "pearson")

cor.test(master_MCI$ab4240,,lm3.res, method = "pearson")

cor.test(master_AD$ab4240,,lm4.res, method = "pearson")

cor.test(master_UD$ab4240,,lm5.res, method = "pearson")

cor.test(master$ptau217,lm1.res, method = "pearson")

cor.test(master_CS$ptau217,lm2.res, method = "pearson")

cor.test(master_MCI$ptau217,lm3.res, method = "pearson")

cor.test(master_AD$ptau217,lm4.res, method = "pearson")

cor.test(master_UD$ptau217,lm5.res, method = "pearson")

cor.test(master$GFAP,lm1.res, method = "pearson")

cor.test(master_CS$GFAP,lm2.res, method = "pearson")

cor.test(master_MCI$GFAP,lm3.res, method = "pearson")

cor.test(master_AD$GFAP,lm4.res, method = "pearson")

cor.test(master_UD$GFAP,lm5.res, method = "pearson")

########### Mediation analyses ###########

# WMH - GFAP - ptau

model.1 <-lm(ptau217 ~ WMH + site_id, master)

summary(model.1)

model.2 <-lm(GFAP ~ WMH +site_id, master)

summary(model.2)

model.3 <-lm(ptau217 ~ GFAP + WMH + site_id, master)

summary(model.3)

results1 <- mediate(model.2, model.3, treat = "WMH", mediator="GFAP", boot=TRUE, sims=500)

summary(results1)

# GFAP - WMH - ptau

model.4 <-lm(ptau217 ~ GFAP + site_id, master)

summary(model.4)

model.5 <-lm(WMH ~ GFAP + site_id, master)

summary(model.5)

model.6 <-lm(ptau217 ~ GFAP + WMH + site_id, master)

summary(model.6)

results2 <- mediate(model.5, model.6, treat="GFAP", mediator="WMH", boot=TRUE, sims=500)

summary(results2)

# WMH - ptau - NfL resids

model.7 <-lm(lm1.res ~ WMH + site_id, master)

summary(model.7)

model.8 <-lm(ptau217 ~ WMH + site_id, master)

summary(model.8)

model.9 <-lm(lm1.res ~ WMH + ptau217 + site_id, master)

summary(model.9)

results3 <- mediate(model.8, model.9, treat = "WMH", mediator="ptau217", boot=TRUE, sims=500)

summary(results3)

# ptau - WMH - NfL resids

model.10 <-lm(lm1.res ~ ptau217 + site_id, master)

summary(model.10)

model.11 <-lm(WMH ~ ptau217 + site_id, master)

summary(model.11)

model.12 <-lm(lm1.res ~ ptau217 + WMH + site_id, master)

summary(model.12)

results4 <- mediate(model.11, model.12, treat = "ptau217", mediator= "WMH", boot=TRUE, sims=500)

summary(results4)

# GFAP - ptau - NfL resids

model.13 <-lm(lm1.res ~ GFAP, master)

summary(model.13)

model.14 <- lm(ptau217 ~ GFAP, data = master)

summary(model.14)

model.15 <- lm(lm1.res ~ GFAP + ptau217, data = master)

summary(model.15)

results5 <- mediate(model.14, model.15, treat = "GFAP", mediator = "ptau217", boot = TRUE, sims = 500)

summary(results5)

# ptau - GFAP - NfL resids

model.16 <-lm(lm1.res ~ ptau217, master)

summary(model.16)

model.17 <-lm(GFAP ~ ptau217, master)

summary(model.17)

model.18 <-lm(lm1.res ~ GFAP + ptau217, master)

summary(model.18)

results6 <- mediate(model.17, model.18, treat = "ptau217", mediator="GFAP", boot=TRUE, sims=500)

summary(results6)

########### Figure 2 ###########

coplot(ptau217 ~ WMH|GFAP, data = master, panel = function(x, y, ...) {

points(x, y, ...)

abline(lm(y ~ x), col = "blue")})

########### Path analysis (Figure 3) ###########

# Whole sample

model <-'

lm1.res ~ TESTVALUE + WMH + ptau217 + site_id

ptau217 ~ TESTVALUE + WMH + site_id

GFAP ~ WMH + site_id'

fit <- cfa(model, data = master)

summary(fit, fit.measures = TRUE, standardized=T,rsquare=T)

fit <- cfa(model, data=master)

graph_sem(model = fit)

# Cognitively stable

model <-'

lm2.res ~ TESTVALUE + WMH + ptau217 + site_id

ptau217 ~ TESTVALUE + WMH + site_id

GFAP ~ WMH + site_id'

fit <- cfa(model, data = master_CS)

summary(fit, fit.measures = TRUE, standardized=T,rsquare=T)

fit <- cfa(model, data=master_CS)

graph_sem(model = fit)

# MCI

model <-'

lm3.res ~ TESTVALUE + WMH + ptau217 + site_id

ptau217 ~ TESTVALUE + WMH + site_id

GFAP ~ WMH + site_id'

fit <- cfa(model, data = master_MCI)

summary(fit, fit.measures = TRUE, standardized=T,rsquare=T)

fit <- cfa(model, data=master_MCI)

graph_sem(model = fit)

# Dementia

model <-'

lm4.res ~ TESTVALUE + WMH + ptau217 + site_id

ptau217 ~ TESTVALUE + WMH + site_id

GFAP ~ WMH + site_id'

fit <- cfa(model, data = master_AD)

summary(fit, fit.measures = TRUE, standardized=T,rsquare=T)

fit <- cfa(model, data=master_AD)

graph_sem(model = fit)

########### Supplementary Figure 1 ###########

model <-'

NfL~ TESTVALUE + WMH + ptau217 + site_id

ptau217 ~ TESTVALUE + WMH + site_id

GFAP ~ WMH + site_id

WMH ~ age + site_id'

fit <- cfa(model, data = master)

summary(fit, fit.measures = TRUE, standardized=T,rsquare=T)

fit <- cfa(model, data=master)

graph_sem(model = fit)
