# Supplementary figures and images for: Cerebrovascular disease is associated with Alzheimer’s plasma biomarker concentrations in adults with Down syndrome
Source: Brain Commun. 2024 Sep 25;6(5):fcae331. doi: 10.1093/braincomms/fcae331 (PMC11472828; doi:10.1093/braincomms/fcae331)

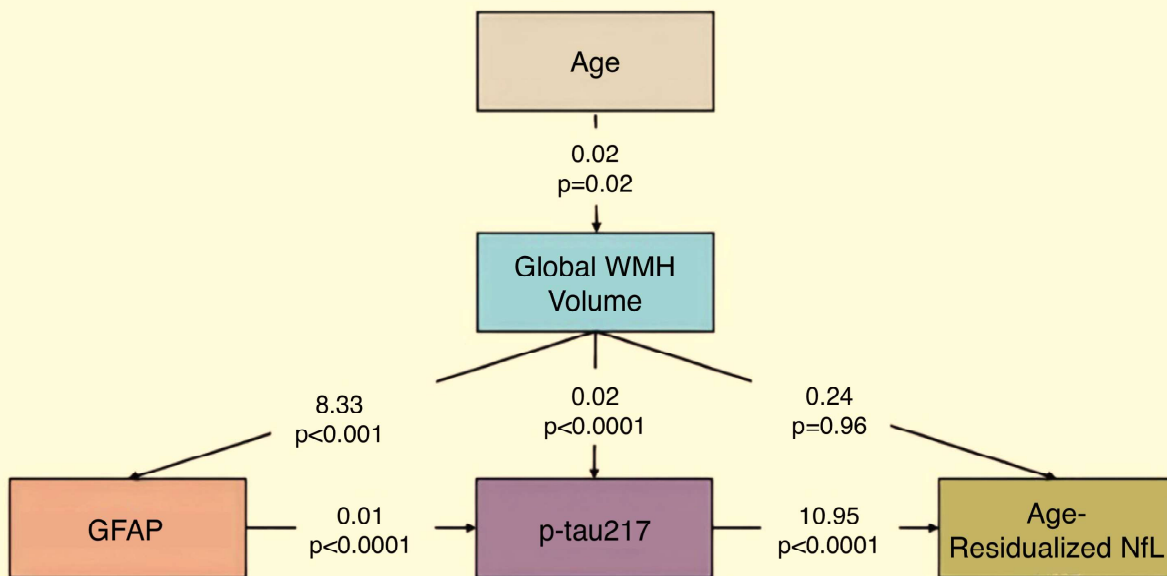

Supplement: fcae331_Supplementary_Data [file fcae331_supplementary_data.zip › Supplementary_Figure_1.pdf]
